# Retention of HIV infected pregnant and breastfeeding women on option B+ in Gomba District, Uganda: a retrospective cohort study

**DOI:** 10.1186/s12879-018-3450-9

**Published:** 2018-10-24

**Authors:** George Kiwanuka, Noah Kiwanuka, Fiston Muneza, Juliet Nabirye, Frederick Oporia, Magdalene A. Odikro, Barbara Castelnuovo, Rhoda K. Wanyenze

**Affiliations:** 1 Department of Health Policy Planning and Management, Makerere University School of Public Health, P.O Box 7072, Kampala, Uganda; 2 Department of Epidemiology and Biostatistics, School of Public Health, Makerere University College of Health Sciences, Kampala, Uganda; 3 Department of Disease Control and Environmental Health, Makerere University School of Public Health, Kampala, Uganda; 4 Department of Research, Infectious Diseases Institute, Makerere University College of Health Sciences, Kampala, Uganda

**Keywords:** EMTCT, PMTCT, Retention, LTFU, HIV, Option B+

## Abstract

**Background:**

Lifelong antiretroviral therapy for HIV infected pregnant and lactating women (Option B+) has been rapidly scaled up but there are concerns about poor retention of women initiating treatment. However, facility-based data could underestimate retention in the absence of measures to account for self-transfers to other facilities. We assessed retention-in-care among women on Option B+ in Uganda, using facility data and follow-up to ascertain transfers to other facilities.

**Methods:**

In a 25-month retrospective cohort analysis of routine program data, women who initiated Option B+ between March 2013 and March 2015 were tracked and interviewed quantitatively and qualitatively (in-depth interviews). Kaplan Meier survival analysis was used to estimate time to loss-to-follow-up (LTFU) while multivariable Cox proportional hazards regression was applied to estimate the adjusted predictors of LTFU, based on facility data. Thematic analysis was done for qualitative data, using MAXQDA 12. Quantitative data were analyzed with STATA® 13.

**Results:**

A total of 518 records were reviewed. The mean (SD) age was 26.4 (5.5) years, 289 women (55.6%) attended primary school, and 53% (276/518) had not disclosed their HIV status to their partners. At 25 months post-ART initiation, 278 (53.7%) were LTFU based on routine facility data, with mean time to LTFU of 15.6 months. Retention was 60.2 per 1000 months of observation (pmo) (95% CI: 55.9–64.3) at 12, and 46.3/1000pmo (95% CI: 42.0–50.5) at 25 months. Overall, 237 (55%) women were successfully tracked and interviewed and 43/118 (36.4%) of those who were classified as LTFU at facility level had self-transferred to another facility. The true 25 months post-ART initiation retention after tracking was 71.3% (169/237). Women < 25 years, aHR = 1.71 (95% CI: 1.28–2.30); those with no education, aHR = 5.55 (95% CI: 3.11–9.92), and those who had not disclosed their status to their partners, aHR = 1.59 (95% CI: 1.16–2.19) were more likely to be LTFU. Facilitators for Option B+ retention based on qualitative findings were adequate counselling, disclosure, and the desire to stay alive and raise HIV-free children. Drug side effects, inadequate counselling, stigma, and unsupportive spouses, were barriers to retention in care.

**Conclusions:**

Retention under Option B+ is suboptimal and is under-estimated at health facility level. There is need to institute mechanisms for tracking of women across facilities. Retention could be enhanced through strategies to enhance disclosure to partners, targeting the uneducated, and those < 25 years.

## Background

Progress against HIV/AIDS over the last 15 years has inspired the global commitment to eliminate mother-to-child transmission of HIV (eMTCT) by 2020 [1], and the HIV epidemic by 2030 [2]. Since 1995, an estimated 1.6 million new HIV infections amongst children have been prevented due to the provision of antiretroviral medicines to women living with HIV during pregnancy or breastfeeding. Despite this achievement, many children are still being infected, and dying from AIDS-related illnesses [2].

Mother to child transmission (MTCT) of HIV is defined as the transmission of HIV from an HIV positive mother to her child during pregnancy, labor, delivery or breastfeeding [3]. In June 2013, the World Health Organization (WHO) recommended initiation of antiretroviral therapy (ART) for all pregnant and breastfeeding women with HIV, and continuation of ART for life (Option B+) [4]. Uganda started Option B+ rollout in December 2012. Gomba district was among the first districts to implement Option B+ [5] and had scaled up services to all levels of health facilities by the time this study was conducted. At District level, the Uganda Primary Health care system has four levels of care, including Health Center II, III, IV, and District Hospital. Health center II represents the first level of interface between the formal health sector and the communities and provides only ambulatory services. HC III offers continuous basic preventive, promotive and curative services, with provisions for laboratory services for diagnosis, and maternity care. HC IV provides all the above services including laboratory and comprehensive emergency obstetric services. The district hospital is the referral facility at district level offering specialized services [6].

Retention-in-care along the PMTCT cascade is an important indicator for quality of PMTCT services and a determinant of PMTCT outcomes [7, 8]. Retention-in-care is defined by the WHO as continuous engagement from diagnosis in a package of prevention, treatment, support and care services [9]. Poor retention-in-care is one of the leading causes of virologic failure, drug resistance, and MTCT [10].

It is therefore important that PMTCT retention-in-care is clearly defined, accurately estimated,, and its causes identified and addressed. However, many studies and evaluations that are based on facility data do not accurately estimate retention as they may not account for women who self-transfer to another health facility [11–14]. Without accounting for women who transfer to other facilities, retention-in-care may be under-estimated [15], and the extent of this under-estimation may vary based on the functionality of the referral mechanisms and tracking of patients across facilities. Studies that have attempted to address these gaps have had a short follow-up period and may not show the potential variations in retention during pregnancy, breastfeeding and after cessation of breastfeeding [16]. Further, the underlying barriers to retention have not been well-documented [17].

This study assessed retention-in-care in a 25-month cohort of pregnant and breastfeeding women on Option B+ in Uganda, using facility based data and integrated follow-up to ascertain transfers to other facilities to fully account for retention of women in care. The study also integrated in-depth interviews to explain the outcomes for the women.

## Methods

### Study setting and population

We conducted this study in Gomba district, in central Uganda between May 01st and June 30th, 2017. Gomba is a rural district with 92% of the population rural [18]. The HIV seroprevalence in Gomba is at eight 8.0%, higher than the national prevalence of 6.5% [19] Almost all women in Gomba attend at least one antenatal care visit [20]. The study was a retrospective cohort analysis of routine program data, combined with a mixed methods cross-sectional study. The study population was pregnant and breastfeeding women who started Option B+ between March 2013 and March 2015. We included women who were residents of Gomba for over 6 months and excluded all those who were visitors.

### Data collection methods

Four research assistants were trained to extract the data using a structured abstraction tool. Client charts were reviewed for 1,3,6,12,18 and 25 months attendance, along the eMTCT cascade. The eMTCT cascade is a series of important stepwise events that constitute a vital roadmap to successful eMTCT. The cascade begins with identification of HIV positive pregnant women and ends with the detection of a final HIV status in HIV-exposed infants at 18 months [21].

An attempt to track all the women from the cohort was made, using the telephone contacts and physical addresses of the women which were retrieved from their records. The research assistants called the women for either phone or face-to-face cross-sectional interviews.

A total of 12 in-depth interviews were conducted, with clients purposively selected, including six women that were retained in care at health facility level and six women who were categorized as LTFU. These were deemed sufficient for data saturation given the recurrent themes with no new emerging leads [22]. Two trained research assistants with experience in qualitative inquiry were employed. Potential participants were called 1 week prior to the interview date to schedule the interview. The in-depth interview guide was structured around predefined broad themes, with preset open ended questions focusing on lived experiences of women on Option B+.

The dependent variable was retention in care and LTFU. A mother was defined as retained in care if she returned to the health facility for care, within 60 days of her last scheduled clinic visit. From clinic ART dispensing records, this was the period estimated for a mother to have run out of drugs. Women who were out of care at the time of conducting the interviews (whether or not they had returned into care at any time during the follow-up period) were categorized as LTFU.

Prior to the data collection, the research assistants were trained, and the data collection tools, including the data abstraction tool and the interview questionnaire pilot tested. Daily feedback during the data collection period was received through meetings of the research assistants and the principal investigator, to review challenges and identify solutions.

### Sample size estimations and data analysis

#### Sample size estimation

The sample size was estimated using the online Stats-To-Do sample size calculator for survival analysis [23]. With a type one error (α) of 0.05, power (1 - β) of 0.84, survival rate in group 1 (SR1), which was the proportion of retention in care in adults receiving ART for health reasons (control group) of 0.87 [24], survival rate in group 2 (SR2), which is the anticipated proportion of retention under Option B+ (exposed group) of 0.69 [14], r = ratio of sample size in group 1 / sample size in group 2 (ssiz1/ssiz2) = (0.2), the total sample size was 232 women. However, we reviewed the records for all the women from all Health center II, III, and IV in the district that were enrolled in the 2-year period (*n* = 518), due to anticipated challenges of missing data.

#### Statistical analysis

Data were analyzed with STATA® 13. During univariate analysis, a descriptive analysis was conducted to calculate the proportions of Option B+ patients who died, stopped ART, were retained in care within the same health facility, and who self-transferred or were formally transferred to other facilities. Because our primary outcome is influenced by time along the Option B+ cascade, survival analysis method was used [14, 25]. The retention based on health facility records at 1, 3, 6, 12, 18 and 25 months after ART initiation was assessed using the Kaplan-Meir methods and survival functions were assessed over the 25-month period. The analysis was up to 25 months, because in our operational definition of retention, a mother was retained if she returned for care within 60 days of her scheduled visit, all those who were scheduled for 24 months reviews had until the end of 25 months to return for their visit. Bivariate analysis: Log-rank test of equality was used, for categorical variables to examine the relationships of individual and facility related independent variables with retention. Cox proportional hazards regression analysis was used to analyze all the variables associated with retention, with a *p*-value of 0.05.

For the qualitative data, audio tape recordings were simultaneously transcribed and translated verbatim from Luganda into English. Transcripts were then uploaded into the qualitative analysis software MAXQDA version 12 and data were analyzed following the six steps of thematic approach developed by Braun and Clarke [26].

## Results

**Fig. 1:**
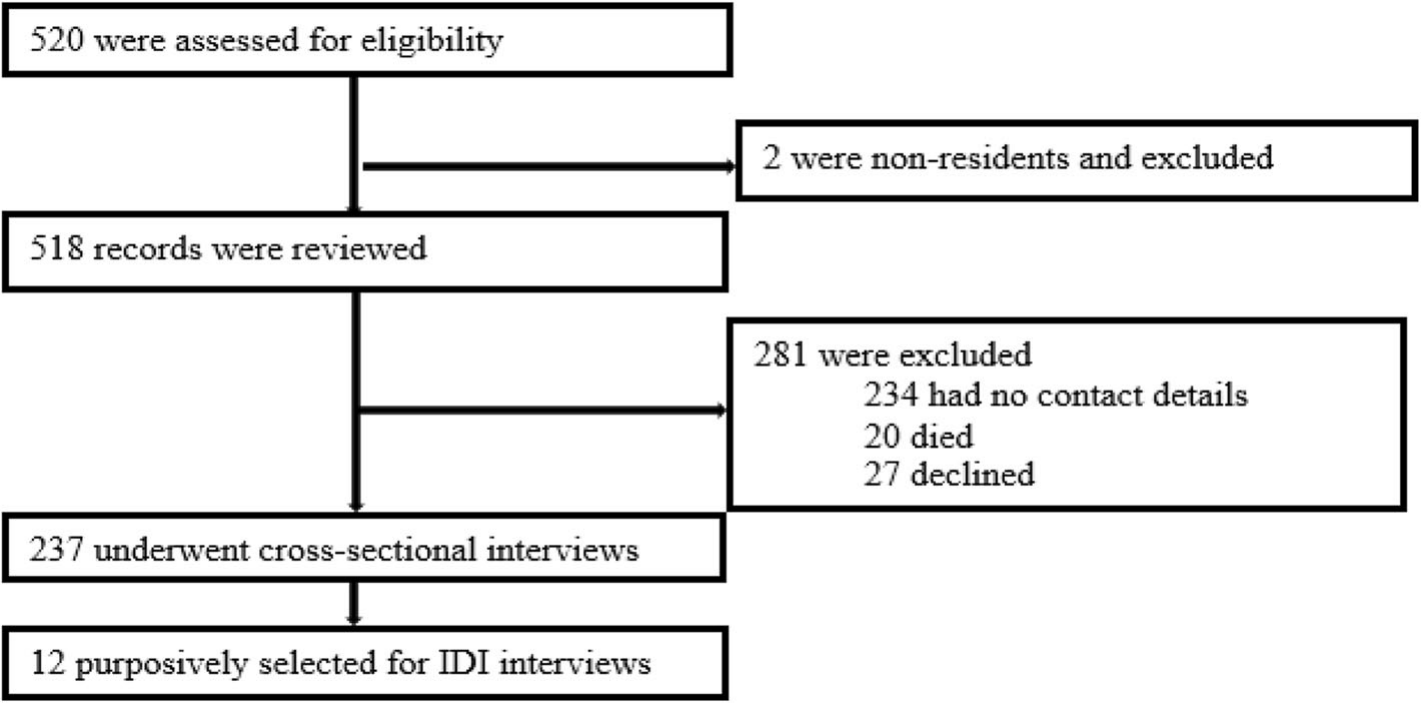
Illustration of the study profile

The study was conducted between 1st May and 30th June 2017. All the records of the 518 pregnant and breastfeeding women who were enrolled in care between March 2013 and March 2015 were included in the 25-month retrospective cohort analysis. Of the 518 women, 237 (46%) were successfully tracked for cross-sectional interviews and ascertainment of retention status; 12 were selected for in-depth interviews **(**Fig. 1**)**. Of the 518 women, 281 (54%) were excluded because 234 (45%) had no phone or residential contacts to enable tracking, 27(10.1%) refused to speak to the interviewers on the phone or physically, and 20 had died.

Nearly half of the women, 234 (45%) were enrolled into Option B+ in 2014, 209 (40.2%) in 2013 and only 77 (14.8%) in 2015. Most of the women (346; 67.2%) started ART during pregnancy while 95 (18.3%) initiated ART before pregnancy, 7 (1.4%) during labor and 68 (13.1%) during breastfeeding. The women were aged between 16 and 48 years, with a mean age of 26.4 (SD: ±5. 5). All the women delivered live babies, and (379; 72.9%) had a DNA PCR test at 6 weeks (Table 1). Most of the women were enrolled at Health Center III, 353 (67.9%) against 167 (32.1) that were enrolled at the Health Center IV. About half of the women 289 (55.6%) had attended primary school, 141 (27.1%) had post-primary education while 90 (17.3%) had no formal education.

**Table 1:** Baseline characteristics of the women from abstracted cohort data

Characteristic	*N* = 518 (%)
Age	
16–24	220 (42.3)
25–48	298 (57.7)
Education Level	
None	90 (17.3)
Primary	287 (55.6)
Post-primary	141 (27.1)
Marital Status	
Single	94 (18.1)
Married/Cohabiting	404 (78.1)
Separated/Divorced	20 (3.8)
Number of children	
0–3	297 (57.5)
4–5	163 (31.4)
5+	58 (11.0)
Health Facility Level	
Health Center II/III	351 (67.9)
Health Center IV	167 (32.1)
Pregnancy Status at Enrollment	
Pregnancy	429 (82.8)
Breastfeeding	89 (17.2)
Disclosure Status	
No	273 (52.6)
Yes	245 (47.4)
Period of starting ART	
Before pregnancy	95 (18.3)
During Pregnancy	348 (67.2)
Labor	7 (1.4)
Breastfeeding	68 (13.1)
Place of Delivery	
Home/TBA^¶^	119 (23.0)
Health Center level II/III^†^	202 (39.0)
Health Center level IV/Hospital	157 (30.3)
Private Clinic	40 (7.7)
DNA-PCR at 6 weeks	
Yes	377 (72.9)
No	141 (27.1)
Final Status of Child†	
Negative	498 (95.8)
Positive	20 (4.2)

^†^The final status of the child at 18 months is done using a rapid antibody test

^†^The Health center II/III are facilities that conduct deliveries but do not provide EMOC services

^¶^Traditional birth attendants provide delivery services in the community, their services

Of the 257 women who were tracked, 20 (7.8%) had died. Among the remaining 237 who agreed to be interviewed, 161 (62.7%) were in care, and 76 (29.6%) not in care. Of the 237 women tracked, 131 (51%) had been classified as LTFU and 126 (49%) as retained, as per facility records. Following analysis of tracking data, true retention was found to be 71.3% (169/237). It was found that 36.4% (43/131) of those initially categorized as LTFU at facility level had not dropped out of care, but had simply self-transferred to another facility,

Using the log-rank test, prespecified subgroups were assessed to determine whether differences in retention at 25 weeks were dependent on participants’ baseline clinical characteristics **(**Table 2**)**. The overall rate of health facility retention was 60.2/1000 pmo (95% CI: 55.9–64.3) at 12 months and 46.3/1000 pmo (95% CI: 42.0–50.5) at 25 months.

**Table 2:** Showing demographic characteristics, perceptions and behavioral practices of women and the sub-group analysis for LTFU following cross-sectional interviews

Characteristic	Retained, *N* = 119 (%)	LTFU, *N* = 118 (%)	*P*-value
Income/Monthly (Uganda Shilling)			
< 50,000	74 (62.2)	92 (77.3)	0.0036
50,001-100,000	27 (22.7)	21 (17.6)	
> 100,000	18 (15.1)	6 (5.1)	
Distance from Health Facility			
< 1 km	19 (16.0)	7 (5.9)	< 0.001
1-5 km	80 (67.2)	60 (50.8)	
> 5 km	20 (16.8)	51 (43.3)	
Health Facility too far^a^			
Disagree	70 (58.8)	29 (24.6)	< 0.001
Agree	49 (41.2)	89 (75.4)	
Difficulty in travelling to Health Facility^a^			
Disagree	70 (58.8)	29 (24.6)	< 0.001
Agree	49 (41.2)	89 (75.4)	
Medicines available⁎			
Disagree	74 (62.2)	24 (20.3)	0.8214
Agree	45 (37.8)	94 (79.7)	
Counselling is sufficient^a^			
Disagree	0 (0)	34 (28.8)	< 0.001
Agree	119 (100)	84 (71.2)	
Peer Mothers help at Option B+ initiation^a^			
Disagree	6 (5)	32 (27.1)	< 0.001
Agree	113 (95)	86 (72.9)	
Difficulty in handling daily hassles^b^			
Disagree	85 (71.4)	51 (43.2)	< 0.001
Agree	34 (28.6)	67 (56.8)	
Difficulty in developing strategies to manage appointments^b^			
Disagree	100 (84)	44 (37.3)	< 0.001
Agree	19 (16)	74 (62.7)	
Difficulty in obtaining support from spouse^b^			
Disagree	80 (67.2)	24 (20.3)	< 0.001
Agree	39 (32.8)	94 (79.7)	
I have confidence in the available HIV care system^b^			
Disagree	0 (0)	6 (5.1)	0.6129
Agree	119 (100)	112 (94.9)	
Disclosed status to spouse?			
No	37 (31.1)	83 (70.3)	< 0.001
Yes	82 (68.9)	35 (29.7)	

^a^Individual attitudes and perceptions about health care services

^b^Personal behavioral practices that influence health services utilization

As shown in Table 3, there were 518 observations at the start with total time at risk of 8080 months. The overall number of women LTFU by 25 months based on the facility data was 278 (53.7%) translating into an overall incidence rate of LTFU of 30/1000 pmo. The mean time to LTFU was 15.6 months, with a minimum exit time of 1 month and a maximum exit time of 25 months. The 25th percentile of the survival time was 3 months while the 50th percentile was 25 months.

**Table 3:** Probabilities of retention in Option B+ over a 24 months period

Time (Months)	No. of women	LTFU	Net^c^ Lost	Retention probability^b^	SE^a^	95% CI^a^	
1	518	94	0	0.82	0.02	0.78	0.84
3	424	42	0	0.74	0.02	0.69	0.77
6	382	40	0	0.66	0.02	0.62	0.70
12	342	30	0	0.60	0.02	0.56	0.64
18	312	38	0	0.53	0.02	0.49	0.57
25	274	34	240	0.46	0.02	0.42	0.51

^a^SE stands for Standard error, CI, for Confidence intervals

^b^The probability of retention is 0.6 by 12 months and 0.46 by 25 months in care

^c^Right censoring was done, with all those who completed the duration of the study censored

**Fig. 2:**
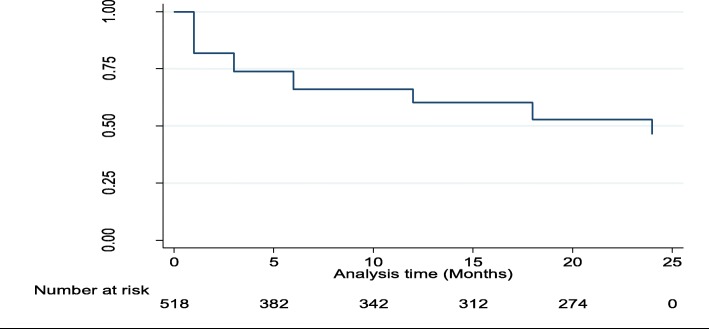
Kaplan Meier for probability of retention over 25 months of follow up

The Kaplan–Meier method was used to estimate the retention curve from the observed times, using facility data. Overall, retention along the whole Option B+ cascade was poor, with the highest risk in the first 6 months **(**Fig. 2**).** Between 18 and 24 months, the critical period of transfer from Option B+ care point to General ART clinic, a considerable proportion of mothers were still lost.

**Fig. 3:**
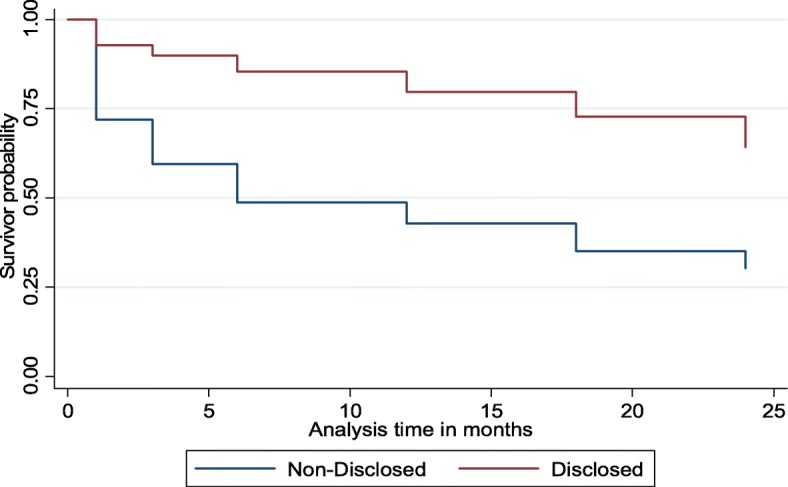
Kaplan Meier showing retention estimates by disclosure status

Figure 3 shows a significant difference between the retention curve for those who disclosed from those who had not disclosed their HIV status. By 25 months post ART-initiation, about 65% of those who had disclosed their status to their partners were retained in care while only 35% of those who had not disclosed were retained (p = < 0.001). Overall, non-disclosure of HIV status to spouse was associated with a risk of LTFU of about 35 percentage points higher than that associated with disclosure (*p* = < 0.001).

At multivariable analysis, women who were less than 25 years were more likely to be LTFU with adj.HR = 1.71; 95% CI: 1.28, 2.30. Those with no education had highest chance of LTFU, aHR 5.55 (95% CI: 3.11, 9.92), primary level of education had aHR 3.83 (95% CI: 2.31, 6.33) in comparison with those who had attained post-primary education (Table 4). Non-disclosure of HIV status was also significantly associated with LTFU, aHR 1.59 (95% CI: 1.16, 2.19). Starting ART during pregnancy, and while breastfeeding was associated with higher chances of LTFU compared to for women who started ART before pregnancy 2.66 (95% CI: 1.60, 4.44).

**Table 4:** Crude and adjusted hazard ratios (HRs) of LTFU in HIV care for Option B+ by socio-demographic characteristics and enabling resources

Characteristic	Crude HR (95% CI)	Adjusted HR (95% CI)	*P*-value
Age(years)			
≤ 24	1.8 (1.43,2,29)	1.71 (1.28,2.30)	< 0.001^a^
≥ 25	1.0	1.0	
Education level			
Post Primary	1.0	1.0	
Primary	3.479 (2.35,5.13)	3.83 (2.31,6.33)	< 0.001^a^
No Formal Education	6.92 (4.51,10.62)	5.55 (3.11,9.92)	< 0.001^a^
Marital status			
Single	1.0		
Married/Cohabiting	0.97 (0.53,1.78)		
Divorced/Separated	1.50 (0.79,2.85)		
Disclosure status			
Yes	1.0	1.0	
No	2.87 (2.21,3.71)	1.59 (1.16,2.19)	0.004^a^
Period of starting ART			
Before Pregnancy	1.0	1.0	
During Pregnancy	3.81 (2.41,6.03)	2.66 (1.60,4.44)	< 0.001^a^
Breastfeeding	3.17 (1.85,5.42)	1.88 (1.02,3.44)	0.042
Place of Delivery			
Home	1.0		
Health Center II/III	0.50 (0.35,0.70)	0.79 (0.54,1.14)	0.209
Health Center IV	0.31 (0.21,0.44)	0.61 (0.41,0.93)	0.021^a^
Private Clinic	1.81 (1.21,2.70)	1.73 (1.08,2.77)	0.021
Baby DNA-PCR at 6 weeks done			
Yes	1.0	1.0	
No	6.77 (5.52,8.76)	4.57 (3.17,6.57)	< 0.001^a^
Results for 6 weeks DNA-PCR received			
Yes	1.0	1.0	
No	2.41 (1.43,4.09)	1.57 (0.79,3.10)	0.192
Final Status of child			
Positive	1.0	1.0	
Negative	1.88 (1.17,3.04)	1.18 (0.63,2.20)	0.602
Income/Monthly (Uganda Shilling)			
< 50,000	3.32 (1.35,8.19)		
50,001-100,000	2.29 (0.86,6.12)		
> 100,000	1.00		
Distance from health facility			
< 1 km	1.00		
1-5 km	1.67 (0.77,3.67)		
> 5 km	3.48 (1.57,7.69)		

^a^Socio-demographic predictors of retention in care for women under Option B+

Analysis of personal behavioral practices revealed that, difficulty in travelling to health facility, aHR 2.30 (95% CI: 1.07, 4.92), and not receiving sufficient counselling, aHR 2.92 (95% CI: 1.49, 5.72) before initiation of ART were the negative predictors of retention under attitudes and perceptions, while presence of peer mothers was a positive predictor (Table 5). Women who had difficulty in obtaining support from the spouse were nearly four times more likely to be LTFU, compared to those who obtained support from their spouses aHR 3.59 (95% CI:2.21,5.81). Those who had difficulty in developing strategies to keep appointments were about two times more likely to be LTFU, aHR 1.89 (95% CI: 1.17,3.05).

**Table 5:** Crude and adjusted hazard ratios (HRs) of LTFU in HIV care for Option B+ by attitudes, perceptions and personal behavioral practices

Characteristic	Crude HR (95% CI)	Adjusted HR (95% CI)	*P*-value
Health Facility too far			
Disagree	1.00		
Agree	2.54 (1.73,4.02)		
Difficulty in travelling to Health facility			
Disagree	1.00	1.00	
Agree	3.58 (2.28,5.62)	2.30 (1.07,4.92)	0.032
Counselling is sufficient			
Disagree	5.12 (3.32,7.87)	2.92 (1.49,5.72)	0.002
Agree	1.00	1.00	
Peer mothers help at Option B+ initiation			
Disagree	2.75 (1.81,4.16)		
Agree	1.00		
Difficulty in handling daily hassles			
Disagree	1.00		
Agree	2.05 (1.42,2.95)		
Difficulty in developing strategies to manage appointments			
Disagree	1.00	1.00	
Agree	3.69 (2.52,5.39)	1.89 (1.17,3.05)	0.009
Difficulty in obtaining support from spouse			
Disagree	1.00	1.00	
Agree	4.53 (2.88,7.13)	3.59 (2.21,5.81)	< 0.001
It’s important to register into HIV care for Option B+			
Disagree	4.29 (2.54,7.23)		
Agree	1.00		
I have confidence in the available HIV care system			
Disagree	3.90 (1.42,10.67)		
Agree	1.00		

### Facilitators and barriers to retention in care for women on option B+

In-depth interviews for the women who were retained and those who were lost showed related issues, therefore, the arrangement of the results incorporates responses for both categories. The unique differences between the two categories are clearly emphasized during the description. Two major themes emerged from the analysis including initiation on Option B+ and adherence to clinic visits/retention in care.

### Facilitators of option B+ initiation

All the women who were retained noted that disclosure, adequate partner counselling and support made their ART initiation ART very comfortable.

*“I really did not face many challenges when starting ARVs because I came to test with my husband, we had talked about the possible outcomes and we were ready for any of them. So, we both came here, and we were both found to be HIV positive. This made it very easy for me because we were counselled together as a couple*” 32-year-old, retained woman.

Other facilitators reported included desire to stay alive and raise HIV-negative babies.

### Barriers of option B+ initiation

Patient unreadiness, inadequate counselling, HIV-related stigma and unsupportive partners and drug side-effects were the issues highlighted mostly by the women, especially those that were LTFU.

*“I was not ready, as soon I tested positive, I was given medicines and was told to swallow them. I asked the health worker for time to think about it, but they said I had to start the medicines immediately. Other people I had heard about started with Septrin but for me I was started on ARVs immediately.”* 23-year-old, LTFU*.*

HIV related stigma was another barrier. Some women feared discrimination and labeling of their children if their HIV status was known and thus felt the need to protect their children.

*“I had the fear of coming to the health facility because I feared meeting my friends here. I would wonder how to fit in society where people knew that I was HIV positive. I feared that they would segregate against my other children, though they were HIV negative”* 26-year-old, LTFU.

### Facilitators of adherence to clinic visits

One mother reported overcoming her daily hassles by better planning and keeping appointments by setting reminders in her phone and memorizing the upcoming appointment date.

*“I have daily work such as farming, cooking would stand in the way of my appointments, but I would just overcome them by better planning such as waking up very early in the morning at 5am and take the cows to the bush for feeding, cook food early before I leave such that by 8am, I am done with all the home chores and I would be in time for my health facility appointment. On the day of appointment, I don’t go for digging. I would remind myself by memorizing and checking my appointment book”* 27-year-old, retained.

Another mother who was retained in care reported that the only reason she managed to stay in care was because she overcame the fear of the negative consequences of disclosure, and she told her spouse about her status and noted that his positive attitude and support helped her adhere to her clinic visits. She however reports that despite his support, he refused to test for HIV, claiming to be immune to the virus.

*“At delivery of the baby, I made up my mind to disclose, and I braced myself for being chased away from home after disclosure. I told him and to my surprise, he said that it was okay and started supporting me. Today he is the one who reminds me to take my ARVs, though for him he says he is immune to the HIV virus and refused to come for testing. Many women are hiding their status from the partners, I encourage them to disclose their status because the experience of living with a supportive partner is needed if one is to have successful care in Option B+”* 28-year-old, retained.

The desire to stay alive was also a strong motivation for mothers to adhere to their care and clinic visits. For most, this was because they wanted to raise their children and see them become independent.

*“I was looking for a healthy life, so that I can live and raise my children. If I don’t take my medicine, I will fall ill and die, and no one will take care of my children”* 27-year-old, retained.

### Barriers of adherence to clinic visits

Side effects were mentioned by all the women who were retained and those who were LTFU. One mother reported that she felt like the side effects of the medicines were going to kill her, so she opted to drop out of care to live a better-quality life.

*“The medicines used to give me a lot of side effects and when I talked to the health workers to change my medicines, they said that I would eventually get used to it, I tried but it became too much, and I stopped. I couldn’t get out of bed, I was sleepless at night and I felt like it was going to kill me even before the HIV/AIDS, so I decided to stop”* 20-year-old, LTFU.

Failure to develop coping strategies was mentioned by about 75% percent of the women who were LTFU. A young mother reported that she had to choose between her employment, her studies and keeping her appointments. She chose the job because she needed the money, and she dropped both out of Option B+ care and school.

*“I was trying to complete my Senior 4 at the time, and I also had to start working to get some money to keep me going. At the salon where I was working, the boss wanted me to work 7 days a week and if I did not work, I would not be paid. I failed to get around this because of the needs I had. My school and work were overwhelming, eventually I had to drop out of school as well, and I did not sit for my O-level exams.”* 20-year-old, LTFU.

Lack of spousal support, coupled with violence and abandonment was another main reason for LTFU. One mother reported that her boyfriend, who had promised to stay with her for life abandoned her when she told him about her HIV positive status. She felt purposeless and dropped out of care.

*“I think I would have tried to stay in care if I had some form of support from my boyfriend. He had promised me that we would be together all our lives, the moment he got to know about my status, he changed. This made me very sad and I couldn’t keep on coming for my appointments because I felt it was purposeless”* 20-year-old, LTFU.

## Discussion

We conducted a study that was a combination of a retrospective cohort analysis and cross-sectional study, investigating retention in care for pregnant and breastfeeding women on Option B+ that were enrolled in care between March 2013 and March 2015, in Gomba district. We found that health facility retention was 60.2% at 12 months and 46.3% at 25 months. However, retention after contact tracing was 10% higher with many women found to have simply transferred to other health facilities. Nearly 50% of women were not traceable, mainly due to lack of contact details. There is a need to establishment a tracking system with reliable contact information and unique identifiers for example through the use of the recently introduced National Identity cards [27, 28]. The main predictors of retention were maternal age, level of education, counselling, disclosure status, timely DNA/PCR testing at 6 weeks and ability to develop strategies to keep appointments. The major facilitators for Option B+ initiation were adequate counselling, disclosure and spousal support, and the desire to stay alive and raise HIV-free children. Drug side effects, inadequate counselling and patient non-readiness, stigma, and un-supportive spouse were among the major barriers to the initiation of lifelong ART. Among the facilitators of adherence to clinic visits, ability to develop strategies to address daily hassles, ability to obtain spousal/family support, and disclosure were the most prominent. The barriers to adherence that were mentioned mostly by both retained and LTFU mothers included, drug side effects, failure to develop strategies to overcome daily hassles, lack of spousal support, and non-disclosure. Several of these barriers are modifiable and could be minimized through adequate counseling and support to enhance preparedness and planning to improve retention.

### Retention in care for pregnant and breastfeeding women

The overall facility retention in care at 25 months, of 46.3% and all other time points was lower than that observed in a study done in northern Uganda [29] and in Malawi [12]. The true retention at 71.3% was similar to what was found in Malawi, in a setting that had electronic medical records system to track self-transfers [14]. High facility LTFU may be explained by poor follow up of women who are LTFU in rural, resource constrained facilities. In rural health facilities, health workers are not motivated enough to be vigilant about LTFU women, as it was found by Kallander [30–33]. It is also noteworthy that about half of the women had no phone or physical contact information, and some had incorrect contact details, which makes any attempt to follow them up virtually impossible. Ensuring complete and accurate contact information is a critical area for intervention [34].

We found that 32.8% of the women were misclassified as LTFU in routine data, underestimating the true level of retention at 25-months. A systematic review and meta-analysis of interventions to improve PMTCT service delivery and promote retention in Tanzania showed that up to 54.4% of LTFU clients had self-transferred to another facility [35]. The lack of a unified database with unique identifiers to help track the patients across facilities is a major challenge to HIV care in general and PMTCT specifically [36].

The rate of health facility LTFU was highest between initiation of ART and the 6-month visit. This may be explained by the failure to navigate the complexity of decisions a woman must make such as immediate initiation on ART [16]. In the second year, a large proportion of women were LTFU around the 18 months period, the critical period of transfer from Option B+ care point to adult care point. This could be explained by the poor health facility linkages between ART clinics and Option B+ care points, and the lack of desire by the mothers to stay on Option B+ once their baby is declared HIV negative [27, 37–40]. Studies have also highlighted the fear to be known as HIV positive and the stigma associated with HIV-only chronic care clinics. The integrated care within the ANC facilities masks the HIV status of the women, which is not possible once they transfer to the predominantly HIV chronic care clinics [41]. This highlights the need to explore integrated service and other models of chronic care for HIV infected pregnant and breast feeding women to minimize LTFU.

Women of a younger age of < 25 years with older spouses had more challenges with retention in care possibly because of their lack of independence from their spouses about decision making, and financial sustenance, which affects their ability to make decisions regarding their health [14, 42, 43]. Older women may have more settled lifestyles with less social pressures, which allow them to better cope with Option B+ care. Women with no or low formal education were also more likely to be LTFU in care possibly due to more challenges in appreciating HIV related information and schedules [43–47].

As documented in other studies, we found that adequate counselling was with a facilitator of retention in care [29, 43]. Pregnant HIV infected women face complex decisions that require major lifestyle and work related adjustments to cope with motherhood and HIV care and thus require counseling and other support to cope [48]. During this transition, support from family and especially their spouses is also crucial to their coping. It is thus not surprising that the women who disclosed their HIV status to the male partners did much better than their counterparts who had not done so [16, 29, 43]. Women are also inclined to provide false contact information because of fear of stigma and discrimination [43]. As documented elsewhere, ability to develop strategies to overcome daily hassles and keep appointments was associated with retention in care [30–32].

Mothers whose babies were not tested for HIV at 6 weeks were more likely to be LTFU. Women who do not adhere to their own care schedules will likely also miss those for their infants [29, 49]. A negative HIV test for the infant could also motivate the women to remain in care [37].

Drug side effects were a major barrier to adherence on Option B+. This may be due to the decreased morale caused by the side effects. These findings are consistent with recent studies in Uganda, Malawi and Ethiopia [16, 40, 42].

### Study strengths and limitations

. The use of qualitative methods allowed for explanatory depth to the quantitative methods. However, there were some limitations. About half of the women were not reached due to lack of contact information—as ably demonstrated through the tracking of women, there is gross underestimation of retention which most likely equally applies to those who were not tracked. Further, the data used was from routine care program records not designed for research purposes, and several desired variables, such as occupation, religion, income, perceptions about eMTCT services were missed during the cohort data abstraction. This was mitigated by tracking and interviewing the women for additional data. Some mothers who were interviewed had to give information on events as far back as 2013, with possibility of recall bias.

## Conclusions

Generally, LTFU was high especially in the first 6 months and during the 12–18-month period (at the time of transfer to chronic care) and was especially more pronounced among the young women with low educational status and those who had not disclosed their HIV status to their partners. Retention in care was also under-estimated at health facility level. Interventions to enhance PMTCT should focus on improving tracking of women across facilities, such as the use of an online electronic medical records system to automatically track self-transfers, and improving counseling and support for disclosure especially among young women and those with low education status.

## Abbreviations

ANC: Antenatal Care
ART: Antiretroviral Therapy
EMOC: Emergency Obstetric care services
EMTCT: Elimination of Mother to Child Transmission
HIV: Human Immunodeficiency Virus
LTFU: Loss-to-follow-up
PMTCT: Prevention of Mother to Child Transmission
WHO: World Health Organization
